# Diagnostic Performance of Perineal MRI–US Fusion Prostate Biopsy: A Single-Center Prospective Cohort Analysis

**DOI:** 10.3390/biomedicines14040797

**Published:** 2026-03-31

**Authors:** Mehmet Gurcan, Yasin Ates, Mert Emre Erden, Rifat Burak Ergul, Ahmet Baris Aydin, Berke Ersoy, Selcuk Erdem, Faruk Ozcan, Oner Sanli

**Affiliations:** 1 Department of Urology, Istanbul Faculty of Medicine, Istanbul University, 34093 Istanbul, Turkey; 2 Department of Radiology, Istanbul University Oncology Institute, Istanbul University, 34093 Istanbul, Turkey; 3 Department of Urology, Division of Urologic Oncology, Istanbul Faculty of Medicine, Istanbul University, 34093 Istanbul, Turkey

**Keywords:** clinically significant prostate cancer, learning curve, magnetic resonance imaging, prostate biopsy, transperineal biopsy

## Abstract

**Background:** Transperineal magnetic resonance (MRI)/ultrasound (US) fusion-guided prostate biopsy has emerged as a promising alternative to the transrectal approach by improving lesion targeting and reducing infectious complications. However, real-world data addressing factors that influence the detection of clinically significant prostate cancer (csPCa), including imaging characteristics and procedural experience, remain limited. **Objective:** To evaluate the diagnostic performance, safety profile, and independent predictors of csPCa detection in patients who underwent transperineal MR/US fusion-guided prostate biopsy, with particular emphasis on PIRADS category, prostate-specific antigen (PSA) level, and procedural learning curve. **Methods:** In this study, patient data were prospectively recorded in a routinely maintained institutional database, while the present analysis was conducted retrospectively. A total of 136 patients with clinical suspicion of prostate cancer—defined as elevated prostate-specific antigen (PSA), abnormal digital rectal examination, or PIRADS ≥3 on multiparametric MRI—underwent transperineal MR/US fusion-guided biopsy between January 2023 and October 2024. **Results:** Prostate cancer was detected in 45.5% of patients, whereas csPCa was identified in 32.3%. The PIRADS category emerged as the strongest independent predictor of csPCa detection, with PIRADS-5 lesions showing a significantly greater likelihood of csPCa than PIRADS-3 lesions (OR 6.70, *p* = 0.006). The PSA level was also independently associated with csPCa detection (OR 1.06 per ng/mL increase, *p* = 0.033). Although csPCa detection rates increased across learning curve groups, procedural experience was not an independent predictor after adjustment. The procedure demonstrated a favorable safety profile, with a low rate of infectious and noninfectious complications despite minimal use of antibiotic prophylaxis. The multivariable model showed moderate explanatory power and acceptable overall classification accuracy. **Conclusions:** Transperineal MR/US fusion-guided prostate biopsy provides reliable detection of clinically significant prostate cancer with a low complication rate and consistent performance across different stages of institutional experience. The PIRADS category and PSA level remain key determinants of csPCa detection, supporting the integration of MRI-based risk stratification into contemporary prostate cancer diagnostic methods.

## 1. Introduction

Prostate cancer (PCa) is the most frequently diagnosed solid malignancy among men and remains a leading cause of cancer-related mortality worldwide [1,2]. Despite advances in risk stratification and imaging modalities, histopathological confirmation through prostate biopsy continues to represent the cornerstone of PCa diagnosis. Traditionally, transrectal ultrasound-guided prostate biopsy (TRUS-Bx) has been the standard diagnostic approach; however, this technique is associated with several well-recognized limitations, including sampling errors, underdetection of anterior tumors, and an inherent risk of infectious complications, ranging from urinary tract infections to life-threatening sepsis [3,4].

The introduction of multiparametric magnetic resonance imaging (mpMRI) has substantially transformed the diagnostic pathway of PCa by enabling lesion-based risk stratification via the Prostate Imaging Reporting and Data System (PIRADS) [5]. Multiple studies have demonstrated that mpMRI-guided targeted biopsy improves the detection of clinically significant prostate cancer (csPCa) while reducing the overdiagnosis of indolent disease [6,7,8,9,10]. As a result, international guidelines now recommend mpMRI prior to biopsy in patients with clinical suspicion of PCa [11,12].

Parallel to these advances, the transperineal approach to prostate biopsy has gained increasing attention as a safer and more comprehensive alternative to the transrectal route. By avoiding rectal wall traversal, transperineal biopsy significantly reduces infectious complications and allows improved access to anterior and apical regions of the prostate, which are frequently undersampled with TRUS-Bx [13,14,15,16]. When combined with MRI–ultrasound (MR/US) fusion technology, transperineal biopsy enables precise targeting of mpMRI-visible lesions while maintaining systematic sampling of the entire gland [17].

Moreover, international guideline updates emphasize the importance of mpMRI prior to prostate biopsy, recommending MRI-based targeted and perilesional sampling strategies to optimize csPCa detection [18]. The transition from systematic, nontargeted biopsies to MRI-informed pathways has been shown to not only improve detection rates but also reduce unnecessary procedures and complications [19].

Although accumulating evidence supports the diagnostic accuracy and safety of transperineal MR/US fusion-guided biopsy, real-world data addressing factors that influence csPCa detection—including PIRADS category, PSA level, patient age, and procedural learning curve—remain limited. In particular, concerns regarding the potential impact of institutional experience and learning curve on oncological outcomes may represent a barrier to widespread adoption of this technique [20]. Furthermore, few studies have evaluated the predictive performance of multivariable models incorporating imaging and clinical parameters within transperineal biopsy cohorts.

In this prospective cohort study, we aimed to evaluate the diagnostic performance of transperineal MR/US fusion-guided prostate biopsy in patients with clinical suspicion of PCa. Specifically, we assessed cancer detection rates, pathological outcomes, complication profiles, and independent predictors of csPCa, with particular emphasis on the role of PIRADS category, PSA level, and procedural learning curve. Additionally, we evaluated the classification performance of a multivariable regression model to further characterize the predictive value of these parameters in contemporary prostate cancer diagnostics.

## 2. Materials and Methods

Patient data were prospectively recorded in a routinely maintained institutional database, while the present analysis was conducted retrospectively. All procedures were performed as part of routine clinical practice; therefore, the requirement for ethical approval was waived due to the retrospective nature of the study. Written informed consent was obtained from all patients before biopsy. Patient data were analyzed anonymously.

Between January 2023 and October 2024, a total of 136 patients who presented to our department with clinical suspicion of prostate cancer (PCa defined by elevated prostate-specific antigen (PSA) levels, abnormal digital rectal examination findings, or a prostate imaging reporting and data system (PIRADS) score ≥3 on multiparametric magnetic resonance imaging (mpMRI)) underwent transperineal MRI/ultrasound (MR/US) fusion-guided prostate biopsy.

Multiparametric MRI examinations were performed prior to biopsy using a 1.5-T MRI scanner without the use of an endorectal coil. MRI images were interpreted by experienced radiologists according to the Prostate Imaging Reporting and Data System (PIRADS). Suspicious lesions identified on mpMRI were subsequently targeted during biopsy using MRI–ultrasound fusion guidance [21]. The number of targeted biopsy cores varied depending on the size and number of MRI-visible lesions, according to the discretion of the biopsy performer.

Patients were eligible for inclusion if they had clinical suspicion of prostate cancer defined by elevated prostate-specific antigen (PSA) levels, abnormal digital rectal examination findings, or a PIRADS score ≥3 on multiparametric magnetic resonance imaging. Patients undergoing confirmatory biopsy during active surveillance were also included. Prostate volume was not considered a selection criterion for performing the transperineal biopsy. Patients with PIRADS <3 lesions or those unable to undergo MRI were managed according to standard clinical practice (conventional TRUS-guided biopsy or clinical follow-up) and were not included in the present study. Thirty-seven patients had undergone a prior prostate biopsy before enrollment; 22 of these patients were under active surveillance for previously diagnosed low-risk prostate cancer, while 15 had had a prior negative biopsy. Meanwhile, 11 patients were receiving dutasteride therapy. No correction for PSA levels was applied in the analyses because the duration of dutasteride use was not documented.

Patients were excluded if they had contraindications to MRI or prostate biopsy, had active urinary tract infection at the time of biopsy, or had incomplete clinical or pathological data.

All mpMRI examinations were reviewed and reported by a single experienced genitourinary radiologist to ensure consistency in lesion identification and PIRADS assessment. Transperineal MR/US fusion-guided prostate biopsies were performed via the BK5000 Fusion^®^ system (BK Medical, Herlev, Hovedstaden, Denmark). All biopsy procedures were performed by two experienced uro-oncologists. One surgeon had approximately 20 years of experience with TRUS-guided prostate biopsy and had performed more than 3000 procedures, while the second surgeon had approximately 10 years of experience with more than 2000 TRUS biopsies. Both surgeons had prior experience with transperineal MRI–US fusion biopsy and had been performing this technique for approximately 22 months during the study period.

Clinical parameters including age, PSA level, digital rectal examination findings, PIRADS score, prostate volume, PSA density, lower urinary tract symptoms, and prior biopsy history were prospectively recorded. Biopsy-related variables—such as preoperative antibiotic prophylaxis, anesthesia method, total number of biopsy cores obtained, number of targeted cores taken from mpMRI-defined index lesions, amount of extraprostatic tissue sampled, and postprocedural complications—were also documented. Pathological findings were retrieved from a prospectively maintained database and analyzed via descriptive statistical methods. 

## 3. Statistical Analysis

Statistical analyses were performed via IBM SPSS Statistics software (IBM Corp., Armonk, NY, USA). Continuous variables were assessed for normality via visual inspection of histograms and distribution characteristics. Normally distributed variables are expressed as the means ± standard deviations, whereas nonnormally distributed variables are summarized via appropriate descriptive statistics. Categorical variables are presented as frequencies and percentages.

Clinically significant prostate cancer (csPCa) was defined as an International Society of Urological Pathology (ISUP) grade > 1 and was used as the primary outcome variable for subgroup and regression analyses.

Comparisons of categorical variables, including csPCa detection rates across learning-curve groups, were performed using the Pearson chi-square test. Continuous variables were compared between multiple groups via the Kruskal–Wallis test when appropriate.

To evaluate independent predictors of csPCa detection, multivariable logistic regression analysis was conducted. The variables included in the regression model were selected based on clinical relevance and the prior literature and included the learning curve group, PIRADS category, prostate-specific antigen (PSA) level, and patient age. Model performance was assessed via omnibus tests of model coefficients, Nagelkerke R^2^ statistics, and classification accuracy. The results of the regression analysis are reported as odds ratios (ORs) with corresponding *p* values.

The discriminative performance and diagnostic accuracy of the multivariable model was evaluated with Receiver Operating Characteristic (ROC) curve analysis. The Area Under the Curve (AUC) was calculated with corresponding 95% confidence intervals (CI) to determine the model′s ability to correctly classify clinically significant cases.

A two-sided *p* value of <0.05 was considered statistically significant for all analyses.

## 4. Results

All patients had a mean age of 64.08 ± 7.65 years (range: 38–84), and the mean PSA level was 9.60 ± 12.78 ng/mL (range: 0.34–100). Lower urinary tract symptoms (LUTS) were present in 74.2% (101/136) of the cohort. Abnormal digital rectal examination findings were identified in 88 patients (64.7%). According to the mpMRI results, 36 patients (26.6%) were reported as having PIRADS-3, 77 (57%) as having PIRADS-4, and 22 (16.3%) as having PIRADS-5.

The mean prostate volume was 64.8 ± 35.1 mL (range: 5–181), and the mean PSA density was 0.185 ± 0.296 ng/mL^2^ (range: 0.002–2.8). The mean total number of biopsy cores obtained was 17.3 ± 2.7 (range: 4–26), whereas the mean number of targeted cores taken from mpMRI-defined lesions was 5.66 ± 2.4 (range: 2–17). The mean number of extraprostatic tissue fragments was 1.69 ± 2.5 (range: 0–16), corresponding to 215 extraprostatic cores out of 2353 total cores (9.1%).

A history of prior prostate biopsy was present in 37 patients (27.2%), 22 of whom (16.1%) underwent biopsy while under active surveillance. Until April 2023, 18 of 26 patients (13.2%) received preoperative fluoroquinolone prophylaxis; however, none of the 116 patients who underwent biopsy between April 2023 and July 2024 (86.8%) received any antibiotic prophylaxis.

General anesthesia was administered to 103 patients (75.7%), spinal anesthesia to 18 (13.2%), sedation/analgesia to 11 (8%), and perineal block to 4 patients (2.9%).

Pathological examination revealed prostate cancer (PCa) in 62 patients (45.5%). Clinically significant prostate cancer (ISUP grade >1) was identified in 44 patients (32.3%). Clinical T-stage was determined based on digital rectal examination findings. Among the 62 patients diagnosed with prostate cancer, 16 (25.8%) were classified as cT1c and 46 (74.2%) as cT2. Among patients diagnosed with prostate cancer, 18 (29%) were classified as low-risk, 32 (51.6%) as intermediate-risk, and 12 (19.4%) as high-risk disease based on pathological Grade Group categories. Among patients diagnosed with PCa, 18 (28.5%) had Grade Group (GG) 1 disease, 20 (31.7%) had GG2, 12 (19%) had GG3, 7 (11.1%) had GG4, and 5 (7.9%) had GG5 adenocarcinoma. One patient was diagnosed with small cell carcinoma on pathological examination. Given the distinct biological behavior and histopathological characteristics of small cell carcinoma compared with those of adenocarcinoma of the prostate, this case was excluded from subgroup and multivariable analyses.

Twenty-two patients who had previously been followed under active surveillance for Gleason score 3 + 3 disease underwent transperineal MRI-fusion biopsy. On multiparametric MR images, five patients had PIRADS-3 lesions, fourteen had PIRADS-4 lesions, and three had PIRADS-5 lesions. The mean PSA level in this cohort was 6.7 ng/mL. Following transperineal MRI-fusion biopsy, Grade Group 1 disease (Gleason score 3 + 3 = 6) was identified in five patients, Grade Group 2 disease (Gleason score 3 + 4 = 7) was identified in six patients, and Grade Group 3 disease (Gleason 4 + 3 = 7) was identified in three patients, while no malignancy was detected in eight patients.

For the five patients diagnosed with GG5 disease, mpMRI evaluation revealed PIRADS-5 in three patients and PIRADS-4 in two patients. Among the seven patients with GG4 (Gleason score 4 + 4 = 8), two were classified as PIRADS-3 and five were classified as PIRADS-5. Among the 12 patients with GG3 (Gleason score of 4 + 3 = 7), one had PIRADS-3, seven had PIRADS-4, and four had PIRADS-5. Among the 20 patients with GG2 (Gleason score 3 + 4 = 7), three had PIRADS-3, 15 had PIRADS-4, and two had PIRADS-5. Finally, among the 18 patients with GG1 (Gleason score 3 + 3 = 6), four had PIRADS-3, twelve had PIRADS-4, and two had PIRADS-5.

Postprocedural complications included urinary retention in 5 patients (3.6%). One patient (0.7%) required hospitalization due to sepsis. Hematuria occurred in one patient (0.7%) and hematospermia occurred in another (0.7%). No additional complications were observed in the remaining patients (Table 1).

Subgroup analyses were performed to evaluate the relationships between clinically significant prostate cancer (csPCa) detection and the PIRADS category, PSA level, age, and learning curve groups.

### 4.1. Learning Curve Analysis

To evaluate the potential impact of procedural experience, patients were stratified into three equal learning curve groups of 45 consecutive cases each. The proportion patients with csPCa increased progressively across the learning curve groups, from 24.4% (11/45) in the first group to 35.6% (16/45) in the second group and 40.0% (18/45) in the third group. However, this increase did not reach statistical significance (Pearson chi-square = 2.600, df = 2, *p* = 0.273).

Comparative analysis of PSA levels across learning curve groups via the Kruskal–Wallis test revealed no statistically significant differences, indicating that baseline oncological risk profiles were comparable throughout the study period.

**Figure 1:**
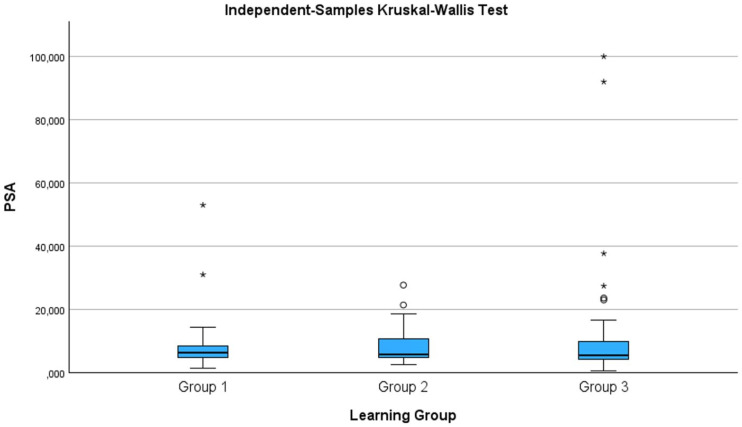
PSA distribution of learning groups.

In addition, the proportion of biopsy cores obtained from extraprostatic tissues decreased significantly with increasing procedural experience. A total of 107 of 744 cores (14.4%) were extraprostatic in the first learning curve group, compared with 66 of 815 cores (8.1%) in the second group and 42 of 793 cores (5.3%) in the third group. Comparative analysis revealed a statistically significant reduction in extraprostatic sampling across learning curve groups (Pearson chi-square = 39.78, df = 2, *p* < 0.001), suggesting improved needle placement accuracy and procedural precision with increasing operator experience (Figure 1).

### 4.2. Multivariate Logistic Regression Analysis

A multivariable logistic regression model was constructed to identify independent predictors of csPCa detection. The model included the learning curve group, PIRADS category, PSA level, and age. The overall model was statistically significant (Omnibus test: χ2 = 36.876, df = 7, *p* < 0.001) and demonstrated an explanatory power with a Nagelkerke R2 of 0.37. Within the adjusted model, PIRADS category emerged as a strong independent predictor of csPCa detection (*p* = 0.019). Within the refined model, patient age emerged as a significant independent predictor (OR 1.10, *p* = 0.012). While PIRADS-5 lesions and higher PSA levels were associated with increased odds of csPCa (OR 3.35 and 1.10, respectively), their independent significance was moderated by the inclusion of PSA density and prostate volume in the multivariable analysis.

The PSA level was also independently associated with csPCa detection, with each 1 ng/mL increase in PSA conferring a 5.8% increase in the odds of csPCa (OR 1.06, *p* = 0.033). Age demonstrated a borderline association with csPCa detection but did not reach statistical significance in the multivariable model (OR 1.06 per year, *p* = 0.072).

The learning curve group was not independently associated with csPCa detection after adjustment for confounding variables (*p* = 0.300).

### 4.3. Model Performance

**Figure 2:**
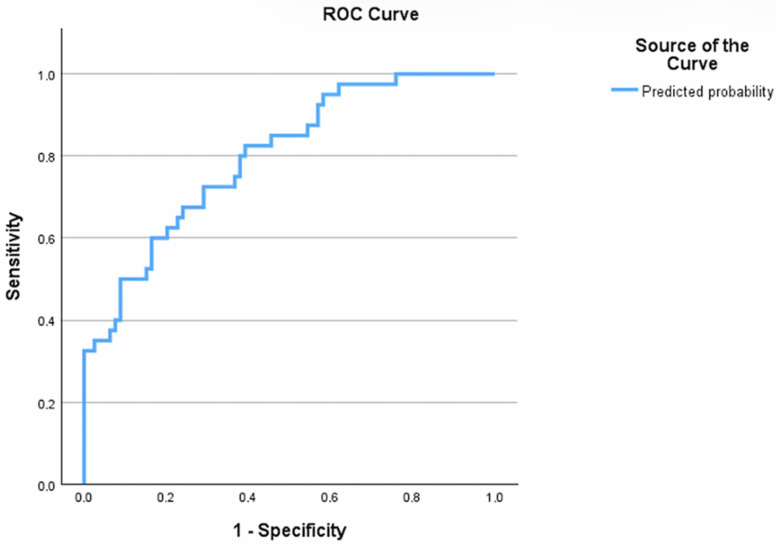
ROC curve of the model.

For discriminative capability of the model, a Receiver Operating Characteristic (ROC) analysis was performed. The model demonstrated good diagnostic performance with an Area Under the Curve (AUC) of 0.799 (95% CI: 0.717–0.881, *p* < 0.001) (Figure 2).

## 5. Discussion

In this study based on prospectively collected clinical data, we evaluated the diagnostic performance and safety profile of transperineal MRI–ultrasound fusion-guided prostate biopsy in patients with clinical suspicion of prostate cancer. Our findings demonstrate that this approach provides reliable detection of clinically significant prostate cancer, with an overall csPCa detection rate of 32.3%, while maintaining a low complication profile. These results support the growing body of evidence favoring transperineal MRI-targeted biopsy as an effective and safe diagnostic strategy in contemporary prostate cancer pathways.

The overall prostate cancer detection rate of 45.5% and csPCa detection rate observed in our cohort are consistent with recently published transperineal MRI–US fusion biopsy series, which reported csPCa detection rates ranging from approximately 30% to 40%, depending on patient selection and MRI risk distribution [9,16]. The relatively high proportion of PIRADS-4 and -5 lesions in our cohort likely contributed to this detection performance and reflects real-world referral patterns in tertiary centers.

Prostate cancer diagnostic pathways have been substantially influenced by the introduction of MRI-targeted biopsy strategies. In a landmark prospective study by Ahdoot et al., which included more than 2000 patients undergoing both MRI-targeted and systematic biopsy, the combination of targeted and systematic sampling significantly improved the detection of clinically significant prostate cancer compared with either technique alone. Importantly, the authors demonstrated that relying solely on MRI-targeted biopsy could result in missed clinically significant tumors, highlighting the continued diagnostic value of combined biopsy strategies. These findings emphasize the importance of integrating imaging-based targeting with systematic sampling approaches in contemporary prostate cancer diagnostics and support the evolving role of MRI-guided biopsy techniques in improving diagnostic accuracy [21].

A key finding of this study is the strong association between the PIRADS category and csPCa detection. In the multivariable analysis, the PIRADS score emerged as the most powerful independent predictor of csPCa, with PIRADS-5 lesions demonstrating a markedly increased likelihood of harboring clinically significant disease compared with PIRADS-3 lesions. This observation aligns closely with prior MRI-based biopsy studies and reinforces the central role of mpMRI-derived lesion characterization in guiding biopsy decisions and risk stratification [7,18]. The lack of statistical significance of PIRADS-4 lesions compared with PIRADS-3 lesions in the adjusted model may be explained by overlap between intermediate-risk lesions and by the limited sample size within subgroups, a phenomenon also reported in previous series [18].

Recent studies have emphasized the value of MRI-based risk stratification combined with PSA-related parameters in improving prostate cancer detection. Zhu et al. evaluated the diagnostic performance of biparametric MRI combined with PSA-derived indicators in patients undergoing transperineal prostate biopsy and demonstrated that PIRADS score and PSA-related markers were significant predictors of prostate cancer and clinically significant disease. In their analysis, PIRADS score together with PSA density emerged as independent predictors of clinically significant prostate cancer, highlighting the importance of integrating imaging findings with clinical biomarkers to refine biopsy decision-making [22].

The PSA level remained an independent predictor of csPCa detection, with increasing PSA values associated with a modest but statistically significant increase in risk, highlighting its continued relevance alongside mpMRI. In contrast, age showed only a borderline association with csPCa, suggesting that imaging and tumor-related factors may outweigh chronological age in predicting clinically significant disease [19].

General anesthesia allows for optimal patient immobility and procedural control, facilitating systematic and extensive sampling and improving intraoperative comfort. Although associated with longer operating room and recovery times, general anesthesia remains a reliable approach, particularly for template-based biopsies or procedures requiring a greater number of cores. In a study comparing transperineal prostate biopsy performed under general and local anesthesia, general anesthesia demonstrated diagnostic outcomes comparable to those of local anesthesia, with similar overall and clinically significant cancer detection rates and low complication profiles [23].

An important aspect of this study was the assessment of procedural learning curve effects. Although csPCa detection rates increased across consecutive learning curve groups, this trend was not statistically significant, and the learning curve group was not an independent predictor in the multivariate analysis. These findings indicate that transperineal MRI–US fusion-guided biopsy provides consistent oncological performance even during early institutional experience when standardized protocols are used, supporting its broader adoption despite concerns regarding operator learning curves [20,24,25].

The safety profile observed in our cohort further supports the advantages of the transperineal approach. Infectious complications are rare, with only one patient requiring hospitalization for sepsis despite the absence of routine antibiotic prophylaxis in the majority of cases. This finding is consistent with the contemporary literature demonstrating significantly lower infection and sepsis rates with transperineal biopsy than with transrectal approaches, even when antibiotic prophylaxis is minimized or omitted [13]. Other complications, such as urinary retention, hematuria, and hematospermia, occurred at low and acceptable rates and were comparable to those reported in recent transperineal biopsy series.

The multivariable regression model demonstrated moderate explanatory power and acceptable classification accuracy. While its performance was high in identifying patients without csPCa, its sensitivity for csPCa detecting was lower, likely reflecting the biological heterogeneity of the disease. Nevertheless, the findings support the value of combining clinical variables with MRI-based risk assessment to inform individualized biopsy decisions.

Several limitations of this study should be acknowledged. First, the single-center design may limit the generalizability of the findings. Second, although mpMRI interpretation was standardized by a single experienced radiologist, interobserver variability could not be assessed. Third, lesion localization within the prostate (anterior versus posterior gland) was not specifically recorded in the dataset, which prevented a separate analysis of diagnostic performance for anterior tumors. Fourth, core-level pathological data distinguishing targeted and systematic biopsy samples were not recorded separately, precluding a detailed comparative analysis of their respective diagnostic yields. Finally, while learning curve effects were explored, the number of cases within each subgroup may have limited the statistical power to detect subtle differences. In addition, the relatively small sample size of the cohort may limit the ability to precisely estimate the true incidence of rare complications, such as sepsis, and therefore the reported safety outcomes should be interpreted with caution.

Despite these limitations, the prospective data, standardized biopsy protocol, consistent imaging interpretation, and comprehensive evaluation of clinical and procedural factors represent key strengths of this study. The inclusion of learning curve analysis and model performance assessment further enhances the clinical relevance of the findings.

## 6. Conclusions

Transperineal MRI–US fusion-guided prostate biopsy offers reliable detection of clinically significant prostate cancer with a favorable safety profile and consistent performance across different stages of procedural experience. The PIRADS category and PSA level remain central determinants of csPCa detection, whereas learning curve effects appear limited when standardized techniques are employed. These findings support the continued integration of transperineal MRI-targeted biopsy into modern prostate cancer diagnostic algorithms.

## Figures and Tables

**Table 1:** Baseline characteristics of the study population.

Variable	Result
Age (years)	64.08 ± 7.65 (38–84)
PSA (ng/mL)	9.60 ± 12.78 (0.34–100)
Digital rectal examination	Normal: 48 (35.3%); Abnormal: 88 (64.7%)
mpMRI PIRADS score	PIRADS-3: 36 (26.6%); PIRADS-4: 77 (57%); PIRADS-5: 22 (16.3%)
Prostate volume (mL)	64.8 ± 35.1 (5–181)
PSA density (ng/mL^2^)	0.185 ± 0.296 (0.002–2.8)
Total biopsy cores obtained	17.3 ± 2.7 (4–26)
Targeted cores obtained	5.66 ± 2.4 (2–17)
Extraprostatic cores/Total cores	215/2353 (9.1%)
Prior prostate biopsy	37 (27.2%)
Biopsy performed during active surveillance	22 (16.1%)
Preoperative antibiotic prophylaxis	18 (13.2%)
Lower urinary tract symptoms (LUTS)	101 (74.2%)
Pathology (PCa detected, n = 62)	
–Gleason 3 + 3 = 6	18 (28.5%)
–Gleason 3 + 4 = 7	20 (31.7%)
–Gleason 4 + 3 = 7	12 (19%)
–Gleason 4 + 4 = 8	7 (11.1%)
–Gleason 4 + 5 = 9/5 + 4 = 9/5 + 5 = 10	5 (7.9%)
Complications	
–Urinary retention	5 (3.6%)
–Fever	0
–Urinary tract infection	0
–Hematuria	1 (0.73%)
–Hematospermia	1 (0.73%)
–Sepsis	1 (0.73%)

## Data Availability

The data presented in this study are available on request from the corresponding author. The data are not publicly available due to patient privacy and ethical restrictions.
